# pH-controlled stacking direction of the β-strands in peptide fibrils

**DOI:** 10.1038/s41598-020-79001-x

**Published:** 2020-12-17

**Authors:** Wei-Hsuan Tseng, Szu-Hua Chen, Hirotsugu Hiramatsu

**Affiliations:** 1 Department of Applied Chemistry and Institute of Molecular Science, National Chiao Tung University, Hsinchu, 30010 Taiwan; 2 Center for Emergent Functional Matter Science, National Chiao Tung University, Hsinchu, 30010 Taiwan

**Keywords:** Biophysical chemistry, Peptides

## Abstract

Peptides provide a framework for generating functional biopolymers. In this study, the pH-dependent structural changes in the 21–29 fragment peptide of β_2_-microglobulin (β_2_m_21–29_) during self-aggregation, i.e., the formation of an amyloid fibril, were discussed. The β-sheet structures formed during parallel stacking under basic conditions (pH ≥ 7.7) adopted an anti-parallel stacking configuration under acidic conditions (pH ≤ 7.6). The parallel and anti-parallel β-sheets existed separately at the intermediate pH (pH = 7.6–7.7). These results were attributed to the rigidity of the β-sheets in the fibrils, which prevented the stable hydrogen bonding interactions between the parallel and anti-parallel β-sheet moieties. This observed pH dependence was ascribed to two phenomena: (i) the pH-dependent collapse of the β_2_m_21–29_ fibrils, which consisted of 16 ± 3 anti-parallel β-sheets containing a total of 2000 β-strands during the deprotonation of the NH_3_^+^ group (p*K*_a_ = 8.0) of the β-strands that occurred within 0.7 ± 0.2 strands of each other and (ii) the subsequent formation of the parallel β-sheets. We propose a framework for a functional biopolymer that could alternate between the two β-sheet structures in response to pH changes.

## Introduction

Extensive research has been conducted on the development of peptide-based functional polymers. Peptides are convenient 1D biopolymers for sequence designs as they can be easily synthesized. So far, remarkable applications of peptides have been reported on the medical functions^1,2^, tissue engineering^3,4^, electrical conductivity^5,6^, the mechanical stability^7,8^, and so on. Some peptides form a self-assembly (e.g., amyloid fibrils)^9–11^. Formation of the self-assembly is an efficient way to fabricate the ordered built-up structures, which has been found as interesting materials in various research fields.

When establishing a strategy for designing amyloidogenic peptide sequences that exhibit particular functions, it is important to precisely determine the structures and factors that define peptide configurations in amyloid fibrils. In these cases, structural elucidation is often executed using techniques such as solid-state NMR spectroscopy and X-ray crystallography. Vibrational spectroscopy has also been employed for the analysis of the proteins and peptides^12,13^. Recent studies on amyloid fibril structures featured the use of resonance Raman spectroscopy (RR) to analyze poly(Gln) in solution and after self-aggregation^14,15^, Raman microscopy to identify the domain of the human islet amyloid polypeptide responsible for fibrillation^16^, Raman^17,18^ or IR or 2D IR spectroscopy^19^ to investigate α-synuclein, IR and VCD spectroscopy for monitoring the self-assembly of Glu-containing peptides^20^, and 2D IR spectroscopy to distinguish the fibril and oligomer of amyloid β^21^.

We have studied the configuration of the amyloid fibril peptide using vibrational spectroscopy^22–25^. One particularly curious fragment, namely, the 21–29 fragment, of β_2_-microglobulin (β_2_m_21–29_) [^21^NFLNCYVSG^29^] was identified^26^. The amyloid fibril of this peptide (referred to as fAβ_2_m_21–29_) contained β-sheet structures in which the strands were aligned in the parallel β (Pβ) or the anti-parallel β (APβ) conformation depending on the prevailing pH^27^. To the best of our knowledge, there are few reports about peptides capable of changing the direction of their β-strands in the stable form under different circumstances (the stacking direction of the β-strands in amyloid fibrils may change depending on a sequence of short segments; Aβ_1–40_ forms Pβ^28^, whereas its fragments generate APβ^29^, and the structures such as amyloid β^30^ and β_2_-microglobulin^25,31^ are heavily influenced by the conditions under which elongation occurs). The intermolecular interaction in the β-strands was compared for the Pβ and APβ structures of fAβ_2_m_21–29_ by monitoring the low-frequency vibration with the low-frequency Raman spectroscopy^32^. We noted that the low-frequency vibrational mode exhibited a more significant force constant in the Pβ structure. Additionally, vacuum–ultraviolet circular dichroism spectroscopy (VUVCD) revealed the main chain structure and the side-chain interactions in the Pβ structure^33^ and demonstrated the nature of the inter-strand aromatic side-chain interactions experimentally. One persistent question about the structure of fAβ_2_m_21–29_ is the origin of the strong pH dependence of the β-strands’ stacking direction in the β-sheets^27^. The pH dependence disappeared after the terminal charges were blocked; this was attributed to the protonation/deprotonation of the terminal groups.

In this study, the influence exerted by the prevailing pH conditions on the β-sheet structures was investigated in detail. The Raman microscope enabled an analysis of the local distribution of the β-sheet structures, and it detected the co-existing two β-sheet structures at the intermediate pH. Also, the β-sheet mixing did not occur even if the two peptides having the distinct preference of the structure (β_2_m_21–29_ for APβ and β_2_m_21–29_–CONH_2_ (β_2_m_21–29_Am) for Pβ) at pH 6.5 were mixed. Based on these observations, we discussed the origins of the exclusive preference in the stacking direction and the pH dependence, the factors influencing the co-existence of the Pβ and APβ structures, and the absence of mixed β-sheets in the two observed stacking directions.

## Results

### pH-dependent structural change

**Figure 1:**
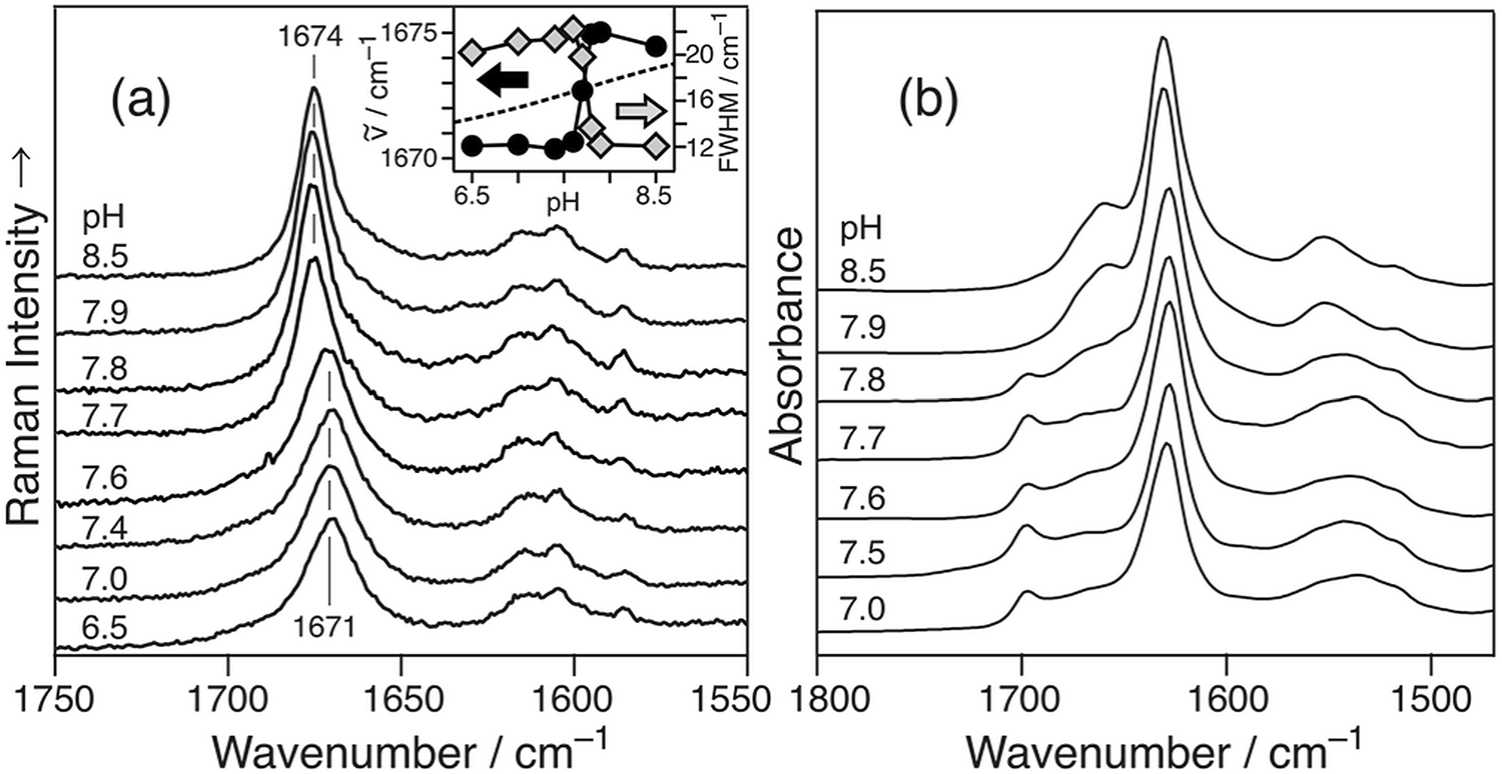
(**a**) Averaged Raman spectra of fAβ_2_m_21–29_ at different pH values. The intensity was normalized with the Raman band of phenylalanine (Phe) at 1003 cm^–1^. The inset shows the pH-dependence of the peak position (left axis), the full width at half maximum (FWHM) (right axis), and the pH-dependence of the protonation/deprotonation equilibrium as calculated using the Henderson–Hasselbalch equation (broken). (**b**) The pH dependence of the IR spectrum of fAβ_2_m_21–29_ at pH 8.6 (top) to 7.0 (bottom) [reproduced and modified with permission from Ref. ^27^].

Figure 1a shows the Raman spectra of fAβ_2_m_21–29_, which was measured at pH 6.5–8.5. The peak position and bandwidth (FWHM) of the Amide I (AmI, the C=O str) band were ca. 1671 and ~ 20 cm^−1^ at pH ≤ 7.6, and 1674 and ~ 10 cm^−1^ at pH ≥ 7.7, respectively. The spectral pattern changed at a pH of 7.6–7.7. The observed spectral change was attributed to the changes in the β-sheet structures (APβ and Pβ) at pH values of ≤ 7.6 and pH ≥ 7.7, respectively^27^. The peak position and FWHM were plotted against pH (Fig. 1a, inset). The pH dependence curve (dashed line), which was predicted by the Henderson–Hasselbalch equation for acid–base equilibria (HA ⇄ H^+^  + A^–^),

1 $${\mathrm{pH}}={\mathrm{p}}K_{\mathrm{a}}+log\frac{\left[{\mathrm{A}}^{-}\right]}{\left[\mathrm{H}\mathrm{A}\right]}$$

did not define the observed pH dependences of the peak position (closed circle) and bandwidth (gray square), suggesting that the observed pH dependence was not due to the protonation/deprotonation of the N-terminal amino group of the peptide monomer. This spectral change was also observed in the IR spectra (Fig. 1b)^27^ (the discrepancy in the pH value associated with the structural changes is explained in Supplementary).

### Raman microscopy at the intermediate pH

**Figure 2:**
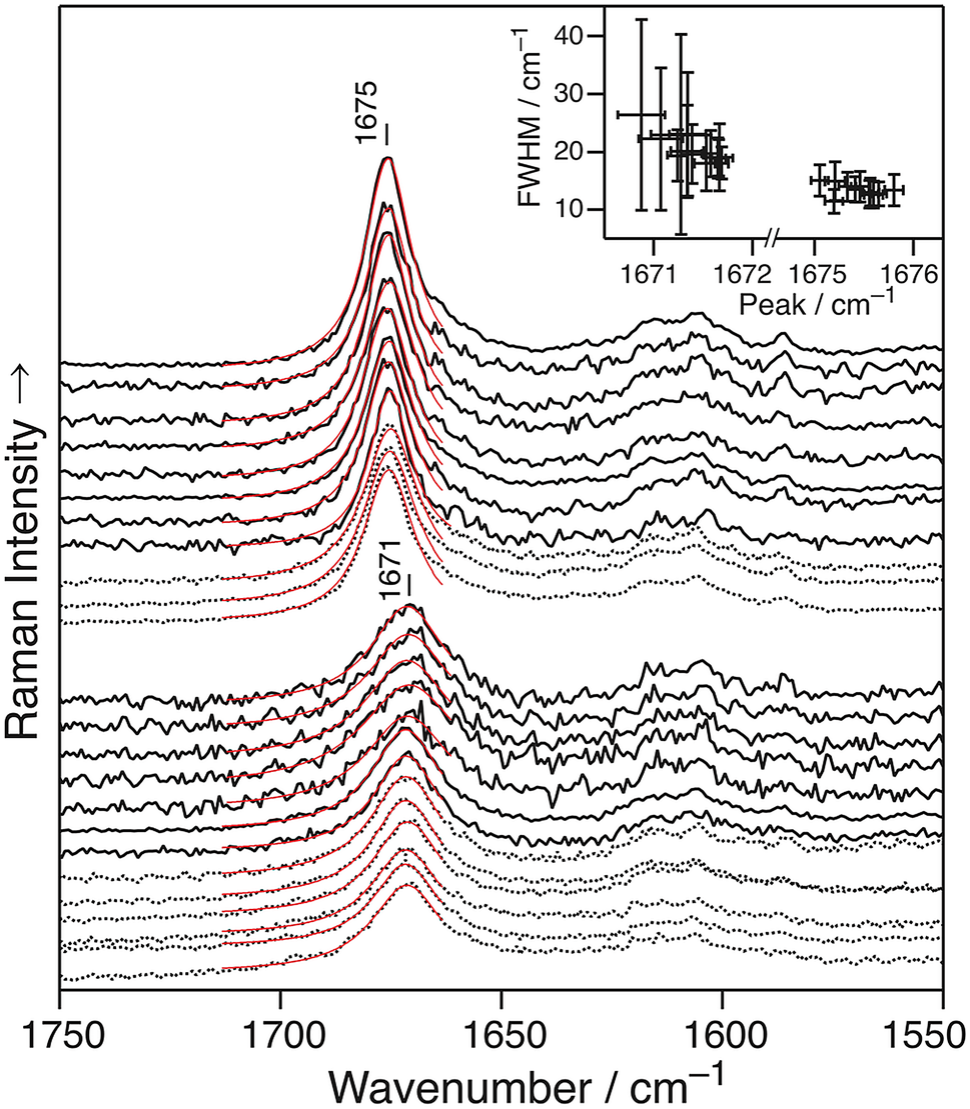
Two types of Raman spectra for fAβ_2_m_21–29_ at pH 7.6 (black, dotted), pH 7.7 (black, solid), and the fitted results (red). Inset: FWHM plot against the peak position of the fitted parameters.

The Raman spectra were obtained with a Raman microscope. The spectra of multiple points observed in the specimen were averaged. Figure 2 shows the Raman spectra at pH values of 7.6 (dotted) and 7.7 (solid). The Lorentzian function (red), which was employed for the fitting analysis, revealed the presence of two groups: one group peak at 1675 cm^−1^ (FWHM =  ~ 10 cm^−1^) and the other at 1671 cm^−1^ (FWHM =  ~ 20 cm^−1^) (Fig. 2, inset). These parameters are identical to those observed at pH ≥ 7.7 and ≤ 7.6, respectively. Thus, APβ and Pβ coexisted at pH values of 7.6 and 7.7.

### Structures of fibrils consisting of the peptides that formed the Pβ and APβ structures

**Figure 3:**
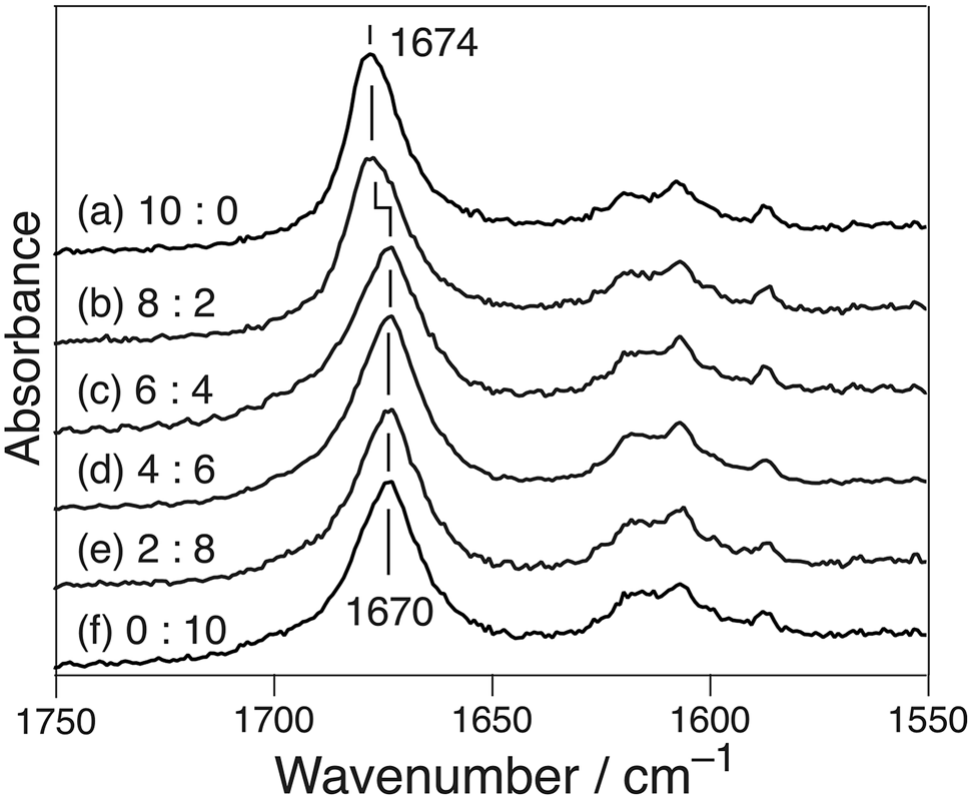
Raman spectra of the amyloid fibrils formed at pH 6.5 from the mixed solution of β_2_m_21–29_Am and β_2_m_21–29_. The respective mixing ratios were (a) 10:0, (b) 8:2, (c) 6:4, (d) 4:6, (e) 2:8, and (f) 0:10.

The peptides, β_2_m_21–29_ and β_2_m_21–29_Am, in which the N-terminal charge was blocked, were mixed at different molar ratios between 10:0 and 0:10 (500 µM), and the amyloid fibrils were prepared at pH 6.5. Based on the peak position, the fibrils of the 100% β_2_m_21–29_ and 100% β_2_m_21–29_Am underwent self-aggregation to form the APβ and Pβ structures at pH 6.5, respectively (Fig. 3). The Raman spectra of the resulting fibrils are also shown therein. The peak position was at 1674 cm^−1^ in the cases of 10:0 and 8:2 mixing ratios, whereas it was 1670 cm^−1^ at 0:10–6:4. The peak position did not shift proportionally with the molar ratios of the two peptides.

### Main peptide chain in the fibrils

**Figure 4:**
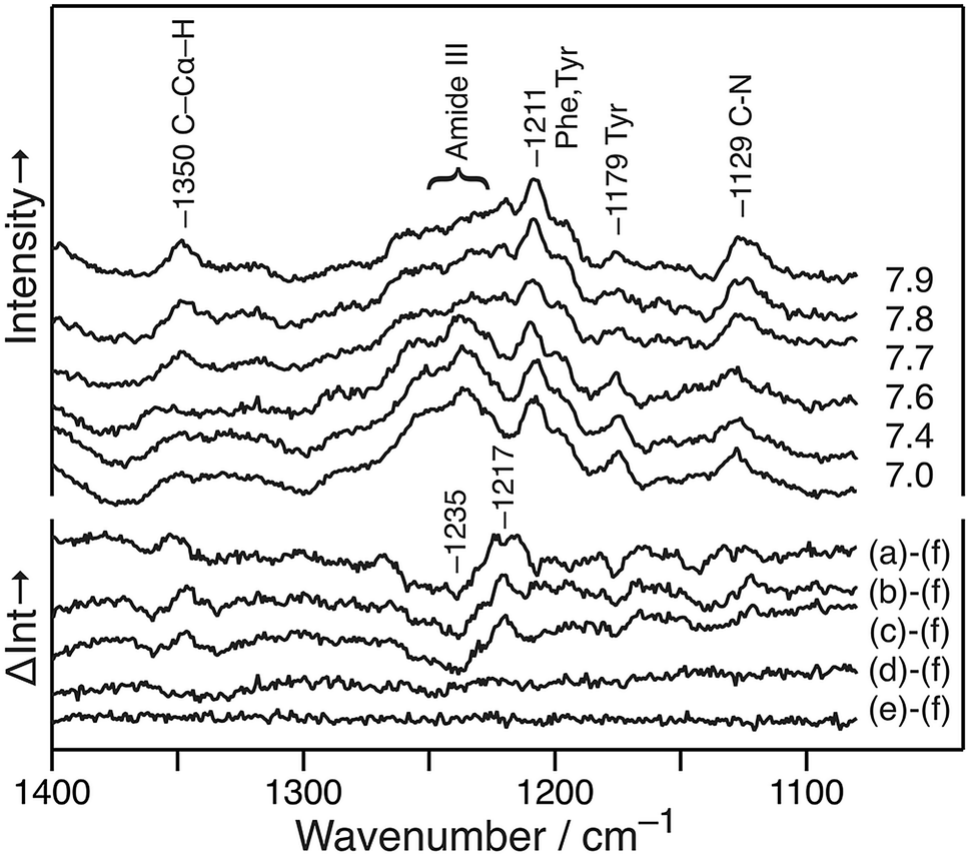
Averaged Raman spectra of fAβ_2_m_21–29_ at pH (a) 7.9, (b) 7.8, (c) 7.7, (d) 7.6, (e) 7.4, (f) 7.0, and their difference spectra from (f).

The lower wavenumber region (1000–1400 cm^−1^) was also analyzed at pH 7.0–7.9 (Fig. 4a–f). The Raman bands at 1131, 1178, 1211, ~ 1239, and 1350 cm^−1^ were obtained at pH ≤ 7.6, and the bands at 1129, 1179, 1211, ~ 1240, and 1350 cm^−1^ were obtained at ≥ 7.7. The differences between each spectrum and that at pH 7.0 are also shown in Fig. 4 (lower). Although no difference was observed at pH ≤ 7.6, peak shifts were observed for the bands at 1129, ~ 1240, and 1350 cm^−1^ at pH ≥ 7.7. These bands were assigned to the C–N stretching (typically observed at 1120–1140 cm^−1^)^34^, the N–H in-plane bending and C–N stretching (Amide III (AmIII)) typically observed at 1230–1245 cm^−1^ for the β-sheet^34,35^, and the vibration of the C–C_α_–H group (typically observed at ~ 1345 cm^−1^)^36^, respectively. The observed peak shift of the main-chain vibrations was ascribed to the difference in the structures of the main peptide chains in Pβ and APβ.

## Discussion

Figure 1 shows the pH dependence of the stacking direction of the fAβ_2_m_21–29_ β-strand. The spectral pattern of the AmI band was binary and corresponded to the Pβ and APβ structures in the IR and Raman spectra, respectively. Figure 2 shows that the Pβ and APβ structures existed separately at pH 7.6–7.7. Their structures were distributed separately because of the inhomogeneity in the reaction tube in which the densities of the two structures differed. Pβ of fAβ_2_m_21–29_ exhibited higher intermolecular vibration than APβ in the position^32^. The larger force constant of Pβ suggested that the distance of the β-strands therein was shorter, i.e., the densities of Pβ and Apβ would not be the same. Additionally, the different packings of the side chains of each β-strands caused the differences in their densities^37^.

**Figure 5:**
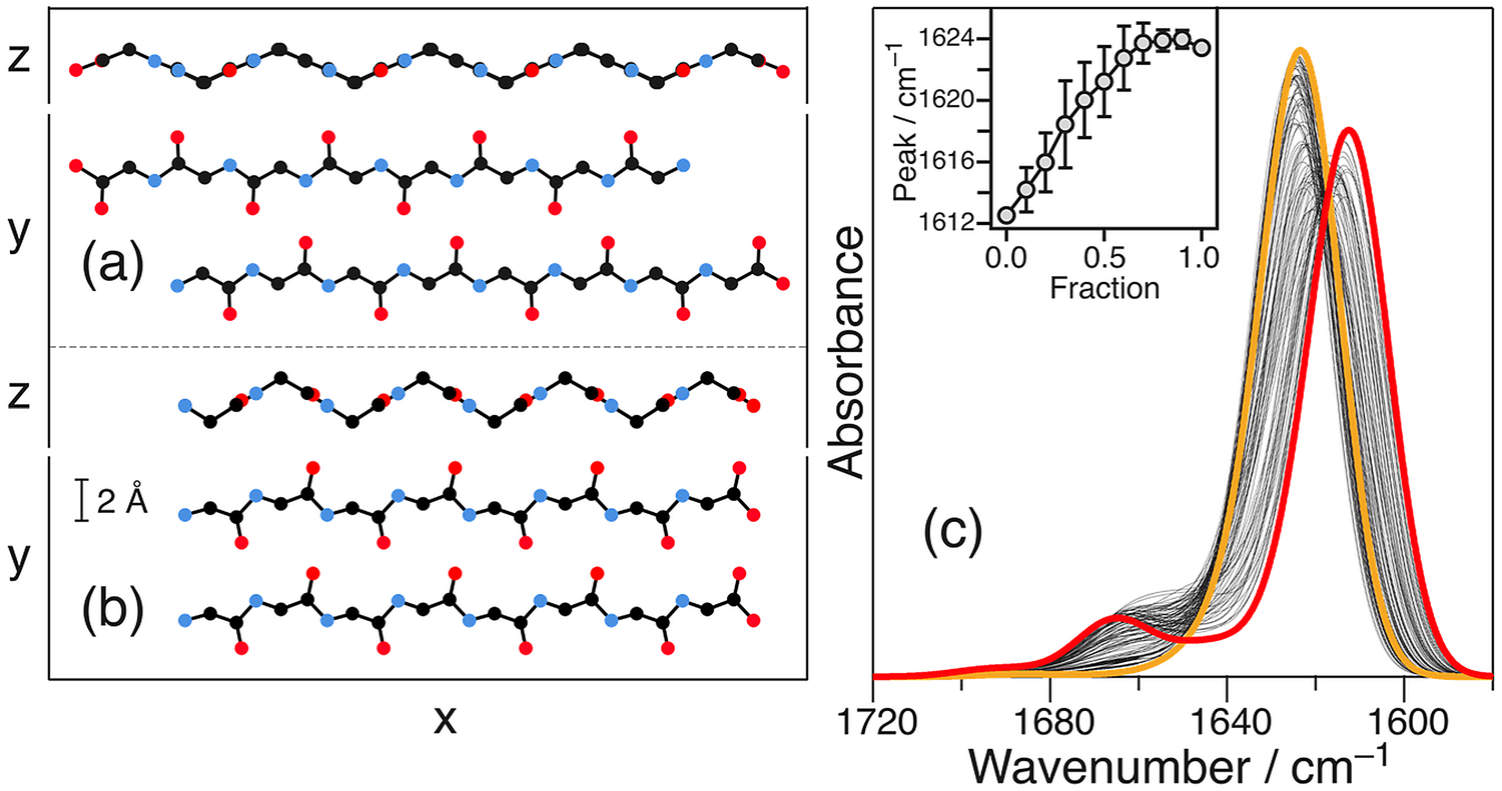
Units (**a**) A and (**b**) B are the models of the anti-parallel and parallel stackings of the two β-strands. The calculated IR spectra of the β-sheet models containing 16 units (32 strands); the fractions of units A and B varies from 10:0 (red) to 0:10 (orange). The inset shows the influence of the fraction on the peak position. The average value calculated for 50 models is plotted, and the error represents standard deviation.

To verify whether the mixed-β structure exhibited characteristic features or not, we calculated the envelope of the AmI band of the mixed-β structure in the IR spectrum by considering the coupling of the transition dipole moments^38^ (see Supplementary). The electrostatic coupling of the transition dipole moments as a function of the orientation and distance of the oscillators^39,40^ caused the collective motions of the oscillators and characteristic peak positions of the AmI bands of each secondary structure^41^. The distribution of the AmI oscillators was determined from structural models consisting of units A (a model of the anti-parallel stacking (Fig. 5a)) and B (a model of the parallel stacking (Fig. 5b)). The dihedral angles {φ, ψ} of the residues were set to {− 139°, 135°} in A and {− 119°, 113°} in B^42^. The bond length was set by GaussView 6^43^ and employed subsequently. The mixed-β structure models were prepared by randomly aligning different mixing ratios of units A and B from 10:0 to 0:10. Each model consisted of 16 units (32 strands). The calculated results (Fig. 5c) suggested that there was a gradual shift in the peak position instead of a binary one. The shift in the binary peak (Figs. 1 and 2) indicated the absence of the mixed-β structure. This absence was also noticed in the β_2_m_21–29_/β_2_m_21–29_Am mixture (Fig. 3). Thus, the β-sheet structures in fAβ_2_m_21–29_ could precisely select the stacking direction.

The selection of the stacking direction was precise because the distance of the C=O and N–H groups were different for the Pβ and APβ structures^42^. The distances between the oxygen atoms of the C=O groups on the same side of the β-strand (the N–C_α_–C–N linkage) were 7.15 and 6.73 Å in APβ (Fig. 5a) and Pβ (Fig. 5b), respectively. The difference was confirmed by the lowered peak shift in the AmIII band (Fig. 4). The frequency of AmIII was markedly dependent on the value of Ψ^44^ and only moderately influenced by φ^45^. The relationship between Ψ and the frequency of AmIII, as derived by Lednev et al., indicated that the decrease in Ψ from 135° (APβ) to 113° (Pβ) lowered the frequency of AmIII by 18 cm^−1^^46^, and the observed peak shift from 1235 to 1217 cm^−1^ correlated with this value. The β-structures were not mixed because the mismatch of these main-chain structures was not compensated for in the regular, rigid β-sheet structures of the amyloid fibrils. The distortion of the APβ structure could shorten the periods of the residue, thereby facilitating stable hydrogen bonding interactions with the parallel β-strand^47^. The mixed-β structure was observed at the edge of the β-sheet, e.g., in ubiquitin (1UBQ), carboxypeptidase A (1YME), and transthyretin (5CN3).

The Pβ and APβ structures were formed separately. Other than the observed preference for the β-sheets, which was due to the presence of F22, L23, C25, Y26, and V27 in the sequence^48^, no strong preference for the stacking direction was expected in the amino acid sequence because the amino acid residues in β_2_m_21–29_ generally appeared in both the Pβ and APβ sheets^49^. The amino acid sequence of β_2_m_21–29_ preferred the Pβ structure when the terminal charges were blocked^27^ because of the absence of the Coulombic force of the terminal charges. Considering the change in the Coulombic interaction, which was due to the protonation/deprotonation of the NH_3_^+^ group, we determined the stability of the APβ structure at a given pH, as described below.

Generally, the amyloid fibril consisted of multiple protofilaments, which contain multiple β-sheets each^9–11^. *m* was set as the number of β-sheets in one fibril*,* and *N* was set as the number of β-strands in each β-sheet. Thereafter, *N* was ~ 2000 for every 1 µm of the protofilament since a β-strand represented a distance of 4.7 Å from one another^50^. Here, *n* ($1 \le n \le N$) was utilized to label each β-strand in one protofilament, i.e., (*m*, *n*) indicated the *n*th strand in the *m*th sheet. In our case, the deprotonation of the NH_3_^+^ group lowered the stability of each APβ sheet. Thus, the fibril structure was destabilized when deprotonation occurred at multiple points of the various β-sheets in proximity. Here we introduced a parameter, *h*, to define the distance from a particular strand in one β-sheet and assume the fibril collapses when the β-strands were deprotonated in all the sheets in proximity, i.e., within *h*. The edges of the β-sheets were not considered in our calculations because *N* was considered to be much larger than *h*. Afterward, we formulated the probability, *P*_fib_, of an APβ fibril collapse as follows:

2 $${P}_{\text{fib}} = {P}_{\text{dep}}\cdot {\left\{1 - {\left(1 -{ P}_{\text{dep}}\right)}^{2h + 1}\right\}}^{m - 1},$$

where *P*_dep_ is the probability of the deprotonation of the NH_3_^+^ group of the *n*′th strand, {1–(1–*P*_dep_)^2* h*+1^} represents the probability of deprotonation in proximity to ± *h* in another β-sheet. The fibrils in all the *m*-sheets collapsed at the *n*′th position when the NH_3_^+^ groups were deprotonated at the (*n*′ ± *h*)th positions. The probability of fibril collapse at a certain point (*P*_col_) was calculated as follows:

3 $${P}_{\text{col}} = 1 - {(1 - {P}_{\text{fib}})}^{N}.$$

Since the same probability applied to any position in the fibrils, a collapse at one point destroyed the fibril in a finite time. Notably, the collapse of APβ competed with the reconstruction of APβ or the construction of Pβ. The Henderson–Hasselbalch equation (Eq. 1) was employed to obtain the probability of the deprotonation of the NH_3_^+^ group, *P*_dep_, as follows:

4 $${P}_{\text{dep}} = \frac{{10}^{pH - pK\text{a}}}{1 + {10}^{pH - pK\text{a}}}.$$

**Figure 6:**
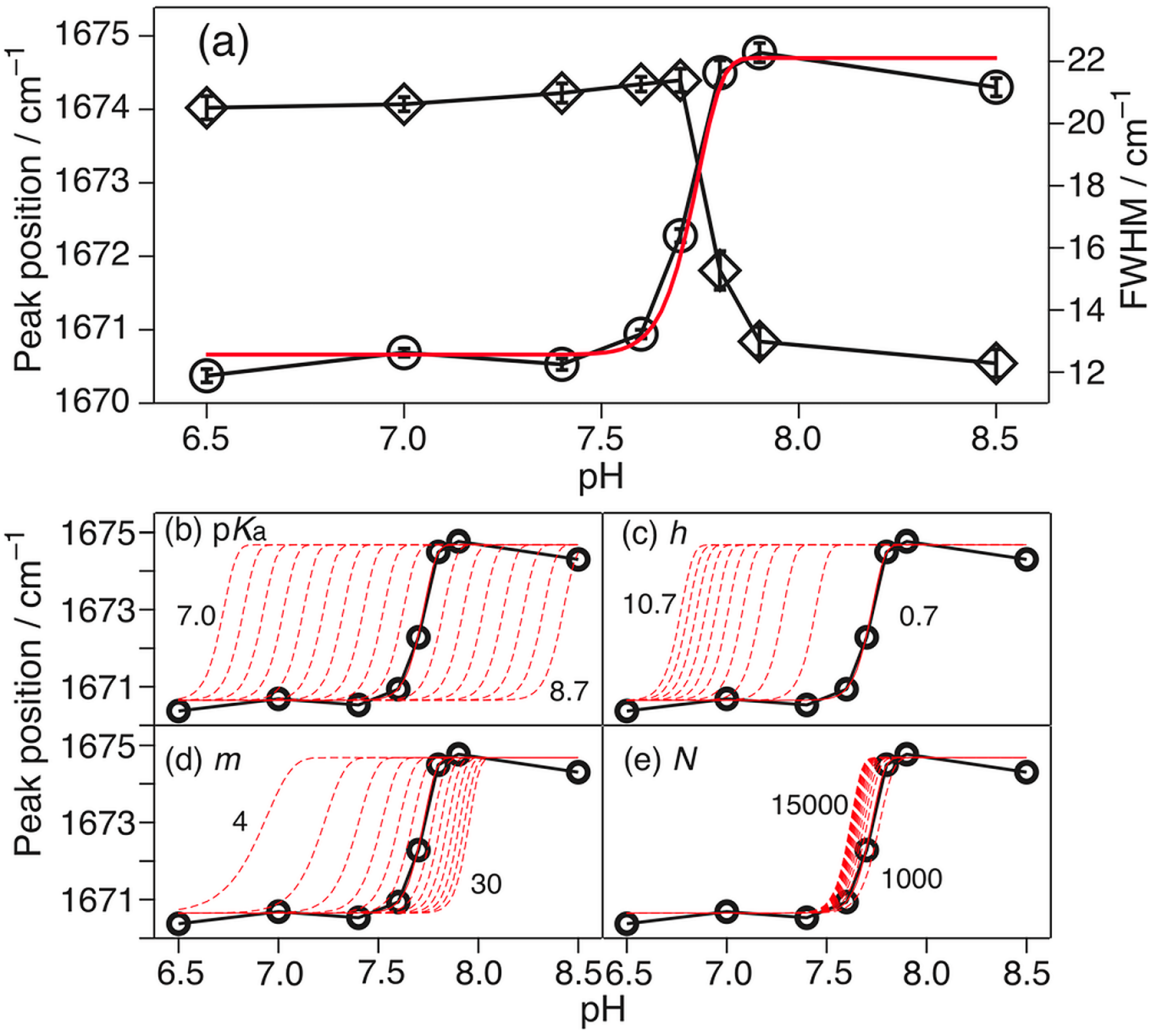
(**a**) Observed pH dependences of the peak position (circle, left axis) and FWHM (diamond, right axis) versus the calculated pH dependence of the probability of APβ collapse employing p*K*_a_(NH_3_^+^) = 8.0 (fixed), *N* = 2000 (fixed), *m* = 16, and *h* = 0.7 (red). The calculated dependence of the probability of APβ collapse employing (**b**) p*K*_a_ (step = 0.1), (**c**) *h* (step = 1.0), (**d**) *m* (step = 2), and (**e**) *N* (step = 1000).

The red line in Fig. 6a was obtained from Eqs. (2)–(4) and the following parameters: p*K*_a_(NH_3_^+^) = 8.0 (fixed)^51^, N = 2000 for a 1 µm fibril (fixed in this work), *m* = 16 ± 3, and *h* = 0.7 ± 0.2. *m* and *h* were selected so that the red curve could fit the pH dependence curve of the peak position. The value of *m* (16 ± 3) indicated that the fAβ_2_m_21–29_ fragment in the APβ structure contained 13–19 β-sheets. Typically, one fibril contains 2–6 protofilaments^52^. The number of β-sheets in one layer of each protofilament was determined to be 4.0 ± 2.1. The presence of multiple β-sheets in the protofilament was consistent with the lateral interactions of the aromatic side chains of fAβ_2_m_21–29_, which was obtained by VUVCD spectroscopy^33^. Thus, the pH dependence of the stacking direction of the β-sheets in the amyloid fibrils could be viewed regarding the discussed results. The influences of each parameter on the pH dependence curve were demonstrated in Fig. 6b–e. Here, the midpoint of the pH dependence curve shifted toward basic conditions as the deprotonation p*K*_a_ of the NH_3_^+^ group increased (Fig. 6b). It shifted toward acidic conditions as *h* increased (Fig. 6c), and toward basic conditions as *m* increased (Fig. 6d). The curve was not readily influenced by *N* values in the range of 1000–15,000 (Fig. 6e). The reduced p*K*_a_ facilitated the deprotonation of the NH_3_^+^ group, and the increased *h* simplified the collapse, i.e., the APβ fibril structure collapsed even at a low pH value where low deprotonation occurred. The increase in *m* further stabilized the structure of the fibril, thereby shifting the midpoint toward more basic conditions than acidic ones. The pH dependence curve was similar within the *N* range of 1000–15,000 because the *N* obtained was adequately larger than the *h*.

This paper described the pH-controlled stacking direction of β-strands and the mechanism by which the amyloid fibrils of the β_2_m_21–29_ peptide were transformed into energetically stable structures. The observed changes in the Pβ and APβ structures is noteworthy; there is an example of the transient conversion of the two β-sheet structures of amyloid-β protein (the occurrence of APβ^53–55^ before the formation of the Pβ fibril)^28^. Regarding pH, there are reports of its effects on the morphology^31,56,57^, and reversibility of the chirality (of the insulin fibril) due to the occurrence of an opposite helical twist without the accompanying transformation of the β-sheet structures^58^. The pH-dependent control of the β-sheet structures could facilitate a novel strategy of imparting functionality to this beneficial biopolymer.

## Conclusion

The β_2_m_21–29_ peptide drastically changed the stacking direction of the β-strand in its sheet structure in a pH-dependent manner. Raman spectroscopy was employed to demonstrate the predominance of the Pβ or APβ structure under certain pH conditions and the corresponding absence of the mixed-β structures. The mixing of the two β-sheet structures was improbable because of the incompatibility of the main-chain structures in the β-sheets of the amyloid fibril. The mechanism proposed in this study featured the pH-dependent control of the fibril structures by destabilizing the APβ structure, which occurred after the deprotonation of the NH_3_^+^ moieties.

## Method

### Sample

A peptide containing β_2_m_21–29_ (^21^NFLNCYVSG^29^) and its analog in which the C-terminal charge was blocked (^21^NFLNCYVSG^29^–NH_2_; β_2_m_21–29_Am) were synthesized in an Initiator^+^ Alstra™ automated microwave peptide synthesizer (Biotage, Uppsala, Sweden). The crude product was purified by a reverse-phase C18 high-performance liquid chromatography (HPLC) column (Cosmosil 5C18-MS-II, Nacalai Tesque, Tokyo, Japan) equipped with an HPLC system (PU-4180-LPG, JASCO, Tokyo, Japan). As determined by mass spectrometry (MS), the monoisotopic mass of the purified peptides corresponded to the calculated values obtained from the amino acid sequences (1015.44), thereby confirming the successful synthesis and purification of the desired peptides. The purified peptides were then dissolved in 0.1 M HCl, lyophilized, and stored as HCl salts at − 30 °C.

### Fibrillation

The peptide was dissolved in dimethylsulfoxide (DMSO) at a concentration of 25 mg/mL (ca. 25 mM) and utilized as the stock solution. The sample solution (2 µL) was mixed with 98 µL of a buffer solution containing 50 mM sodium phosphate and 100 mM NaCl with adjusted pH value. The final concentration of the peptide in the buffer was 500 µM. Next, the sample solution was incubated at 37 °C under quiescent conditions in a water bath for 12 h. The pH value was monitored and controlled during the fibrillation process. After incubation, the solvent containing DMSO was removed from the sample solution before the sample tube was centrifuged at 1.5 × 10^4^ RCF for 10 min at 24 °C. The supernatant (90 µL) was removed, followed by the addition of 20 µL of the buffer. The sedimented fibril chunk was suspended in the buffer by pipetting.

### Raman analysis

Raman spectroscopy was performed with a laboratory-designed confocal Raman microspectrometer^59–61^. Briefly, the 632.8 nm emission of a He–Ne laser (Thorlabs) was introduced to an inverted microscope (Nikon, TE2000-U). The beam (5 mW at the focal point) was focused on the fibril chunks in the suspension (20 µL) in a glass-bottom dish through an objective lens (CFI Plan Fluor; 100× , NA = 1.3, oil-immersion, Nikon). The backscattered light was obtained by the same lens and delivered to an imaging spectrometer (HORIBA Scientific, iHR320) that was equipped with a 1200 grooves/mm grating. Rayleigh scattering was eliminated by an edge filter (Semrock). The Raman signal was detected by a liquid N_2_-cooled charge-coupled device detector (Princeton Instruments; Spec-10:100) with 100 × 1340 pixels operating at − 120 °C. A spectral resolution of 5 cm^−1^ (~ 1 cm^−1^/pixel) was considered as effective. The wavenumber was calibrated utilizing the emission lines of the Ne lamp. The peak positions of sharp Raman bands were reproducible within ± 1 cm^–1^. Each spectrum was recorded at an exposure time of 60 s with ten-times the accumulation. The resulting spectra, which were measured at different points of each specimen (nine points at pH 7.6, 14 points at pH 7.7, and six points at the other pH values), were averaged to obtain the Raman spectrum at each pH. All the measurements were performed at 24 °C. The peak position of each Raman band was derived by fitting the Lorentzian function.

### IR analysis

The IR spectra were recorded on a Fourier-transform IR (FT–IR) spectrometer (Avatar360) that was equipped with an IR microscope (Continuum) (Thermofisher Scientific, MA). The 25 × 25 µm area was measured. Each spectrum was measured by a  1024-accumulation cycle at a spectral resolution of 4 cm^−1^. A pellet of the fibrils, which was prepared via centrifugation was soaked in the buffer solution before it was sandwiched in two CaF_2_ windows. The IR spectrum was measured for the pelleted particles while they were still wet. A reference sample containing the solvent was also measured at a spot that was in proximity to the pellet.

## Supplementary Information

Supplementary Information.
