# Supplementary material for: pH-controlled stacking direction of the β-strands in peptide fibrils
Source: Sci Rep. 2020 Dec 17;10:22199. doi: 10.1038/s41598-020-79001-x (PMC7747703; doi:10.1038/s41598-020-79001-x)
Supplement: Supplementary file 1 — Supplementary Information. [file 41598_2020_79001_MOESM1_ESM.pdf]

Supplementary Information

pH-controlled stacking direction of β-strands in peptide fibrils

Wei-Hsuan Tseng,[1] Szu-Hua Chen,[1] and Hirotsugu Hiramatsu[1,2,]*

1. *Department of Applied Chemistry and Institute of Molecular Science, National Chiao Tung University, Hsinchu 30010, Taiwan,*
2. *Center for Emergent Functional Matter Science, National Chiao Tung University, Hsinchu 30010, Taiwan.*

Table of Contents

1. Error in the pH calibration and its effects

2. Calculation of the Amide I band envelope

1. Error in the pH calibration and its effects

We calibrated the pH meter at three points (pH 4, 7, 10). In the calibration of the pH meter, a deviation in the pH value of the standard solutions as small as 0.05 unit (from the nominal pH values) derives the error of the regression line. According to the regression analysis (Figure S1), the error is ca. 9% and 1.2% for the intersection and slope, respectively, in this case. The estimated error becomes as large as ± 0.13 at pH 7.70. This estimation could explain the discrepancy of the pH values at the intermediate of the structural change in Figs. 1A and 1B, the experimental results that were performed independently.

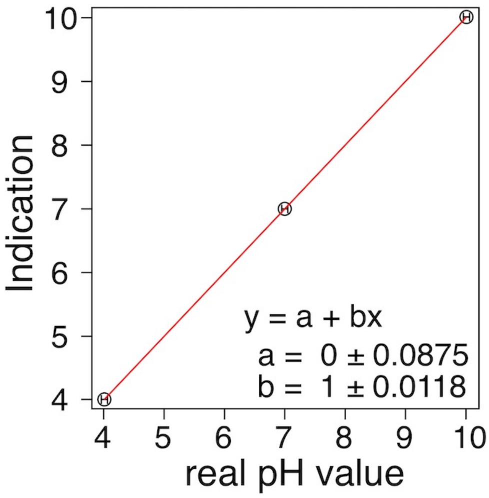

Figure S1 Plot of indicated vs real pH.

It is worth noting that the error of the "pH difference" is smaller than that of the absolute values of the determined pH, because the error of the constant term (i.e., the intersection (the coefficient *a* of the regression line) cancels. The error solely derives from that of the slope (the coefficient *b* of the regression line), and the estimated error is as small as 1.2% for the pH difference. It leads that the step of pH is 0.1 ± 0.001 unit in the pH dependence measurement. This value is accurate enough to conclude that the pH dependent change occurs within the 0.1 – 0.2 pH unit (Fig. 1).

2. Calculation of the Amide I band envelope

We used a truncated procedure of the GF matrix method to analyze vibrational frequencies of amide I modes, as described below.

The GF method for calculating vibrational frequencies of an *N*-atomic molecule requires two $N \times N$ matrices (G and F) representing the kinetic and potential energy of harmonic oscillators, respectively.[1] Eigenvalues and eigenvectors of the GF matrix give the vibrational frequencies and motions, respectively, of the treated vibrational modes. The GF method is practically not applicable to vibrational analyses of large molecules because the calculation is a time-consuming process. Torii and Tasumi have performed a truncated procedure for obtaining GF matrices to calculate the band envelope of the amide I mode (the C=O stretching of the H–N–C=O moiety in the peptide main chain) of proteins.[2]

According to their method, the F matrix is prepared by considering the force constant of the vibrations of the *i*-th amide I oscillators in the sequence as the *ii*-th diagonal term (f_{ii}). f_{ii} of the oscillators in the β-sheet structure was set to 1.625 mdyn Å^{-1} amu^{-1} for all *i*. The dependence of f_{ii} on some factors such as the dihedral angle[3] and hydrogen bonding[4] was not taken into account.

Besides, the vibrational coupling between the *i*- and *j*-th oscillators (f_{ij}, $j \neq i$) was adopted as the *ij*-th off-diagonal term. The off-diagonal *ij* term (f_{ij}) derives from the coupling of the transition dipole moment ($\delta\boldsymbol{\mu}$) of the *i*-th and *j*-th oscillators of the amide I vibration was placed on the C=O bond axis at 0.868 Å from the C atom. The angle between $\delta\boldsymbol{\mu}$ and the C=O bond axis (α) was set to 20°.[2] f_{ij} was calculated by considering the dipole-dipole interaction;

$$f_{ij} = \frac{1}{\varepsilon}\frac{\delta\boldsymbol{\mu}_i \cdot \delta\boldsymbol{\mu}_j - 3(\delta\boldsymbol{\mu}_i \cdot \boldsymbol{n}_{ij}) \cdot (\delta\boldsymbol{\mu}_j \cdot \boldsymbol{n}_{ij})}{R_{ij}^3} \quad (1)$$

where $\delta\boldsymbol{\mu}_x\ [x = i, j]$ is the transition dipole moment of the x-th amide I oscillator (assumed to be 3.70 D Å^{-1} amu$^{-1/2}$), R_{ij} (in Å) is the distance between the i-th and j-th oscillators, and $\boldsymbol{n}_{ij}$ is the unit vector along with the line connecting the two oscillators. ε is the dielectric constant and set to unity. The additional term due to the through-bond interaction[5] was not involved.

The G matrix is set to the unit matrix as each amide I oscillator is regarded to be identical and independent from each other (g_{ii} = 1 and g_{ij} = 0).

We adopted this procedure to calculate the envelope of the amide I band of the Pβ, APβ, and mixed β structures. The structural models for the calculation were prepared as follows; the anti-parallel stacking dimer (unit A in Figure 5(a)) and the parallel stacking dimer (unit B in Fig. 5(b)) were aligned to form the β-sheet. Each β-strand was 5 Å apart from each other. The ratio of the numbers of unit A to B varied from 0 to 1 by 0.1 step. Each dimer unit was allocated randomly, and 50 structures were prepared at each

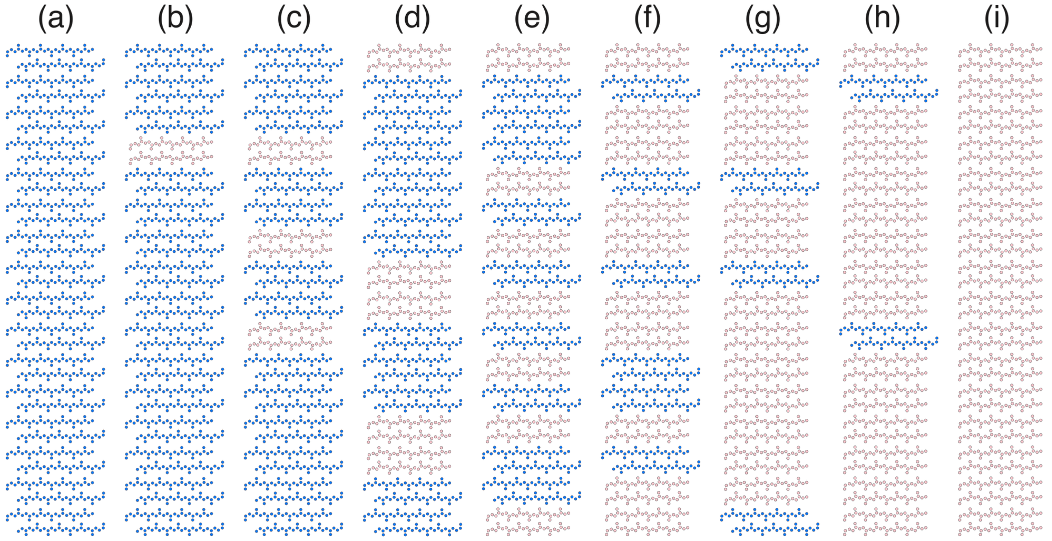

Figure S2 Examples of APβ (a), Pβ (i), and mixed β models (b-h). Ratio between the anti-parallel dimer (blue) and the parallel dimer (pink) is 16 : 0 (a), 15 : 1 (b), 13 : 3 (c), 11 : 5 (d), 9 : 7 (e), 6 : 10 (f), 4 : 12 (g), 2 : 14 (h), and 0 : 16 (i).

condition for the calculation of the amide I envelope. R_{ij} and n_{ij} in Equation (1) were obtained from each β-sheet model (Figure S2 illustrates some examples).

The product of G and F matrices was diagonalized, and the eigenvalues and eigenvectors were obtained. The eigenvalue was converted to the vibrational frequency. The vibrational amplitude of each oscillator and the vibrational frequency are derived from the eigenvector and eigenvalue, respectively. The IR intensity of each mode is calculated from the absolute magnitude square of the sum of the polarization vectors of each Amide I oscillators. The polarization of each oscillator is obtained by multiplying the transition dipole moment vector and the vibrational amplitude.

Each peak of the corrective vibrations of the amide I mode of the β-sheet model was depicted in Figure 5. A Gaussian band with a full-width at half-maximum of 20 cm^{-1} was assumed for each calculated mode, and the envelope of the amide I band was obtained.

References

1 Wilson, J., E. B., Decius, J. C. & Cross, P. C. *Molecular Vibrations*. (Dover Publications, Inc., 1955).

2 Torii, H. & Tasumi, M. Model calculations on the amide-I infrared bands of globular proteins. *J. Chem. Phys.* **96**, 3379-3387 (1992).

3 Ham, S. & Cho, M. Amide I modes in the N-methylacetamide dimer and glycine dipeptide analog: diagonal force constants. *J. Chem. Phys.* **118**, 6915-6922. (2003).

4 Ham, S., Kim, J.-H., Lee, H. & Cho, M. Correlation between electronic and molecular structure distortions and vibrational properties. II. Amide I modes of NMA-nD_2O complexes. *J. Chem. Phys.* **118**, 3491-3498 (2003).

5 Torii, H. & Tasumi, M. Ab Initio molecular orbital study of the Amide I vibrational interactions between the peptide groups in di- and tripeptides and considerations on the conformation of the extended helix. *J. Raman Spectrosc.* **29**, 81-86 (1998).
